# Kin-Aggregations Explain Chaotic Genetic Patchiness, a Commonly Observed Genetic Pattern, in a Marine Fish

**DOI:** 10.1371/journal.pone.0153381

**Published:** 2016-04-27

**Authors:** Jason D. Selwyn, J. Derek Hogan, Alan M. Downey-Wall, Lauren M. Gurski, David S. Portnoy, Daniel D. Heath

**Affiliations:** 1 HoBi Lab, Department of Life Sciences, Texas A&M University–Corpus Christi, Corpus Christi, Texas, United States of America; 2 Marine Science Center, Northeastern University, Nahant, Massachusetts, United States of America; 3 Marine Genomics Laboratory, Department of Life Sciences, Texas A&M University-Corpus Christi, Corpus Christi, Texas, United States of America; 4 Great Lakes Institute for Environmental Research, University of Windsor, Windsor, Ontario, Canada; Department of Agriculture and Water Resources, AUSTRALIA

## Abstract

The phenomenon of chaotic genetic patchiness is a pattern commonly seen in marine organisms, particularly those with demersal adults and pelagic larvae. This pattern is usually associated with sweepstakes recruitment and variable reproductive success. Here we investigate the biological underpinnings of this pattern in a species of marine goby *Coryphopterus personatus*. We find that populations of this species show tell-tale signs of chaotic genetic patchiness including: small, but significant, differences in genetic structure over short distances; a non-equilibrium or “chaotic” pattern of differentiation among locations in space; and within locus, within population deviations from the expectations of Hardy-Weinberg equilibrium (HWE). We show that despite having a pelagic larval stage, and a wide distribution across Caribbean coral reefs, this species forms groups of highly related individuals at small spatial scales (<10 metres). These spatially clustered family groups cause the observed deviations from HWE and local population differentiation, a finding that is rarely demonstrated, but could be more common than previously thought.

## Introduction

Marine organisms with dispersive pelagic larvae are expected to be characterized by little to no genetic differentiation among populations over large geographic areas due to high gene flow. However, many marine species exhibit slight, yet significant, levels of genetic heterogeneity over various local spatial scales [1,2] which may be temporally unstable. Typically in these situations, molecular markers deviate from the expectations of Hardy-Weinberg equilibrium (HWE) within some samples [3–5]. This complex pattern, termed chaotic genetic patchiness (CGP), has been attributed to non-equilibrium conditions caused by variation in reproductive success of breeding adults during larval recruitment [2,6]. By seeking to understand the biological mechanisms underlying these patterns we can deepen our understanding of the ecology and evolution of complex marine populations.

There are several mechanisms that could create CGP across populations of marine organisms [6] including: local habitat differences [1], temporal variability in ocean currents affecting dispersal [7], selection at settlement [8], differential reproductive success [9], genetic drift within isolated populations [2], and the formation of kin-aggregations [2]. In marine populations, kin-aggregations are often overlooked as a mechanism to explain CGP because the probability of sampling relatives is expected to be low for species with highly dispersive pelagic larvae [10]. However, recent studies show that larvae can remain in kin-aggregations in the plankton [7,11,12]. Kin-aggregations likely form during the larval stage through aggregations of locally-spawned eggs and larvae by ocean currents and patchiness of food resources [13]. Various life-history traits, including demersal spawning and shoaling, might also cause kin-aggregation formation in fishes [13].

Here we investigate the population genetic structure of a shoaling marine goby, *Coryphopterus personatus*. Despite being wide-spread throughout the Caribbean little information exists about its life history, particularly information about larval duration, dispersal, and population structure. We seek to use observed patterns of genetic structure to test the kin-aggregation hypothesis and discuss the consequences of this mechanism for the ecology of this and similar species. We then propose competing alternative hypotheses for the mechanism leading to the formation of kin-aggregations.

## Methods

**Fig 1:**
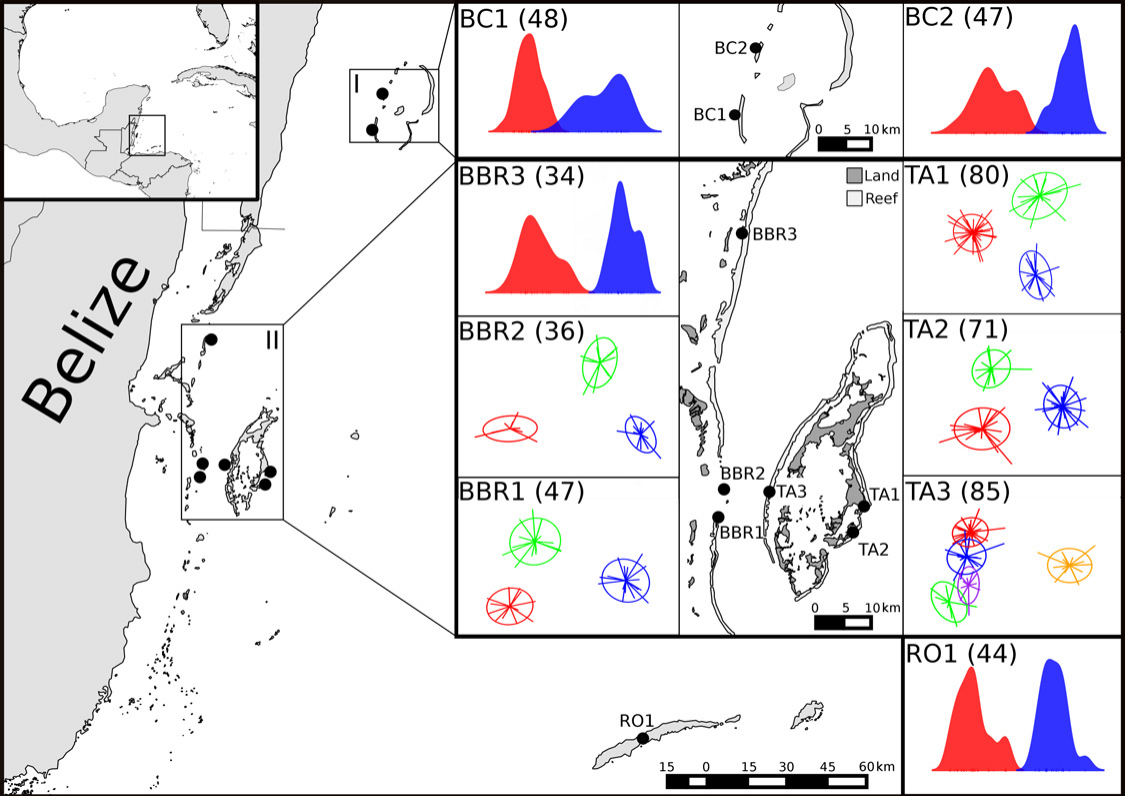
The Mesoamerican Barrier Reef study sites: BC–Banco Chinchorro, Mexico; BBR–Belizean barrier reef, Belize; TA–Turneffe Atoll, Belize; RO–Roatán, Honduras. Insets show scatter plots (and density plots in the case of two clusters) of clusters from DAPC analysis within sampling locations. The axes of the plots are the first two discriminant functions used to delineate clusters with inertia ellipses representing 67% of the variance. The end of the lines connected to the centre of each inertia ellipse represent individuals plotted on each discriminant function and denotes cluster membership. In locations with only two clusters present there is only one discriminant function, as such density plots of proportion of individuals present at each value of the discriminant function were included to show cluster separation. Numbers in parentheses indicate how many individuals were collected from each location.

Fish were collected during summer 2002 from nine sites in the Mesoamerican barrier reef system (34 to 85 per site, Fig 1). Within each site, multiple shoals of *C*. *personatus* were collected by divers from a 1-ha area on the fore-reef and pooled for storage in 95% ethanol. All animals were humanely euthanized in the field using a standard fish sedative, Tricaine mesylate (MS222). Live fish were placed in a seawater bath with MS222 added for 5 minutes or until breathing ceased. All collections were approved by and performed in accordance with the ethical guidelines of the University of Windsor and the Belize fisheries department, the Mexican Secretaría de Medio Ambiente y Recursos Naturales, and the Department of Natural Resources and Environment of Honduras. Genomic DNA was extracted from pectoral fin tissue of each individual following the silica-based 96-well plate protocol of Elphinstone *et al*.[14]. Ten microsatellite loci were chosen from Hepburn *et al*. [15] and Hogan *et al*. [16]. PCR amplification was then performed in 12.5 µL reactions comprised of: approximately 100 ng template DNA, 200 μM of forward dye-label primer and reverse primer, 200 µM of each dNTP, 0.1 U Taq polymerase (0.025 U for CPER 184, Invitrogen, Burlington, Canada), 1x PCR buffer (provided by the manufacturer) and locus specific concentrations of MgCl_2_ and bovine serum albumin (Table 1). PCR conditions were 94˚C for 2 minutes, followed by locus specific numbers of cycles of 94˚C for 15 s, locus specific annealing temperatures and times (Table 1), with a final extension of 72˚C for 90 s. The size of amplicons was determined using a LiCor 4300 DNA Analyzer with GeneImagIR 4.05 software (Scanalytics, Inc).

**Table 1:** PCR Conditions and locus summary statistics.

Locus	Ta (˚C)	Ta (s)	MgCl_2_ (mM)	BSA (mM)	Allelic Richness	H_o_	H_e_	Missing
Cope9 (GACA)_n_	56	15	1.5	0	13	0.403	0.787	0.217
Cope12 (GT)_n_GG(GT)_n_	56	15	2.5	0	9	0.342	0.657	0.150
Cper26 (CA)_8_	46	45	2.5	2	14	0.504	0.715	0.222
Cper92 (TG)_9_	49	15	2.5	0	24	0.278	0.718	0.197
Cper99 (CA)_7_	46	45	2.5	2	10	0.191	0.406	0.063
Cper103 (TG)_7_	46	45	2.5	2	7	0.590	0.619	0.073
Cper119 (TG)_15_	53	15	2.8	0	33	0.570	0.920	0.120
Cper163 (TG)_12_	65–57†	15	2.5	2	19	0.137	0.612	0.258
Cper184 (TC)_19_	65–57†	15	1.25	2	50	0.632	0.834	0.161
Cper188 (CA)_9_	53	15	2.5	0	24	0.392	0.726	0.124

†: indicates touchdown PCR reaction used; Ta (˚C): annealing temperature; Ta (s): annealing time. Missing is the proportion of individuals which failed to amplify at the given locus (complete information on individuals which failed to amplify at each locus and location combination in S1 Table).

One of the common hallmarks of chaotic genetic patchiness is many deviations from Hardy-Weinberg equilibrium combined with weak but significant genetic differentiation between samples. To test for deviations from HWE exact tests for goodness of fit to the expectations of HWE were performed in adegenet [17]. To test the possibility of deviations from HWE being due to elevated levels of inbreeding, Monte Carlo tests for homozygote excess, which is indicative of either high levels of inbreeding, or the presence of null alleles, were performed using the U-score implemented in HWxtest [18]. Inbreeding coefficients were calculated for each site using gstudio [19]. Both inbreeding and null alleles are supported by homozygote excess in a population. Since individuals homozygous for a null allele or heterozygous for two null alleles will present as missing data, there may be an association between the amount of missing data at a locus, in a population and deviation from HWE when null alleles are present. Therefore, we performed a linear regression between the absolute value of the difference between expected and observed heterozygosity and the proportion of individuals which failed to amplify at each locus by sample combination to determine if the observed patterns are consistent with null alleles. To test for genetic differentiation among samples the proportion of different alleles (*P*_*D*_) and pairwise *F*_*ST*_ between sites were calculated across all loci using adegenet [17]. A Mantel test was performed in ade4 to test for isolation-by-distance among sites using *P*_*D*_ and *F*_*ST*_ [20]. Significance was assessed by permuting genotypes among samples 10,000 times, using sequential Bonferroni to correct for multiple testing.

To test the hypothesis that increased levels of relatedness within sites explains the pattern of chaotic genetic patchiness observed here pairwise relatedness (*r*) was calculated between all pairs of individuals using the triadic likelihood estimator, which has been shown to be the least biased relatedness estimator in many circumstances, using the R package related [21,22]. The arithmetic mean of the pairwise relatedness ($\bar{r}$) was calculated for each geographic sample in R v3.1.1 [23]. Significance of mean within sample relatedness was calculated using a one-tail permutation test with genotypes permuted among geographic samples or clusters 1,000 times.

Our initial sampling pooled multiple shoals of individuals; if sites were composed of multiple groups of related individuals (i.e., shoals) and if those groups were pooled during collection, then the mean pairwise relatedness at the site level would be artificially deflated. We performed a Discriminant Analysis of Principal Components (DAPC) to identify genetic clusters of individuals within sites as implemented in adegenet [17,24]. All possible clustering solutions were compared using the k-means clustering algorithm. The number of clusters within each site was found by evaluating all possible values for k and choosing the most likely based on minimum BIC. The mean pairwise relatedness ($\bar{r}$) was then recalculated for each cluster within sites generated with the DAPC and significance determined as above.

As further evidence of elevated relatedness and to minimize error associated with continuous metrics of relatedness, COLONY was used to determine the number of full- and half-sib dyads within clusters using the pair-likelihood score algorithm allowing for inbreeding [25]. We assumed polygamy and polyandry and a 1% genotyping error rate. Sibship was considered when dyads were supported with ≥95% confidence. Evidence of high levels of sibling pairs (both full and half-sibs) within clusters was assessed using a two-tailed permutation test, permuting individuals among clusters 10,000 times. The probability of excluding a group of three unrelated individuals from a full sibship was calculated using code written by the authors [26]. This probability of exclusion is useful in determining the statistical power the set of microsatellite markers used in this study have in determining sibship relationships.

In order to determine if elevated relatedness observed in clusters is the result of multiple dyads of related individuals or a few large groups of relatives within clusters undirected networks were created for each cluster identified using DAPC with individual *C*. *personatus* represented as nodes and sibling relationships (full- and half-sibs) represented as edges [27]. The mean local transitivity, or clustering coefficient, was used to understand the degree of interconnectedness within each cluster. Higher transitivity values indicate more interconnected networks of sibship relationships. Transitivity is a metric used to understand networks and ranges from 0 to 1 with 0 representing no clustering of relationships and 1 representing maximally clustered graphs. Biologically, transitivity values near 0 indicate the presence of multiple related pairs of individuals, which are unrelated to others in the cluster. Transitivity values close to 1 indicate the presence of a few large groups of related individuals. Significance of transitivity within each cluster was assessed by simulating 10,000 random graphs using the Erdos-Renyi model with the same number of vertices (individuals) and edges (sibling connections) as the observed cluster [27,28]. Networks were analysed using the package igraph [29].

## Results

**Fig 2:**
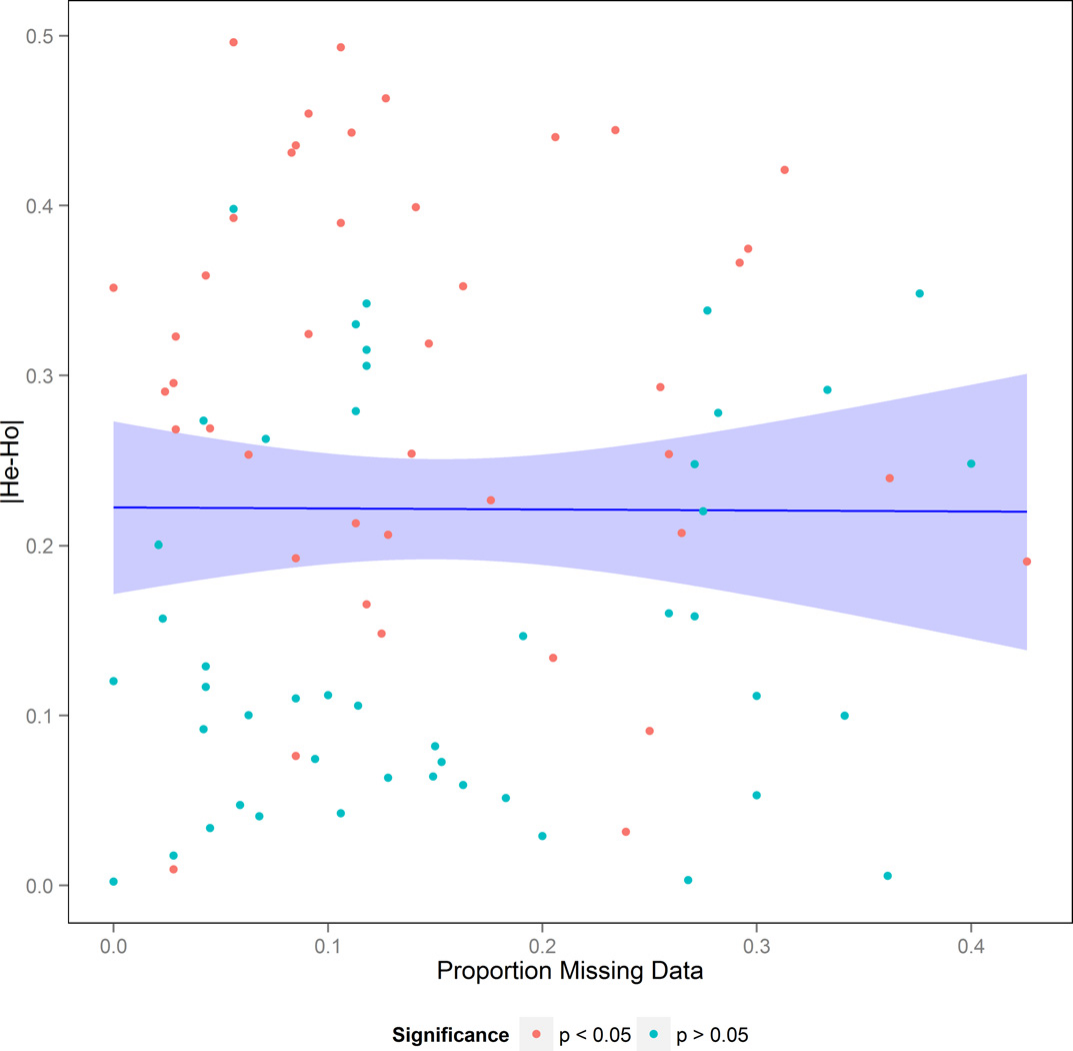
Regression plot showing locus specific sample proportion of individuals which failed to amplify plotted against the absolute value of the difference between expected and observed heterozygosity. The plotted line is the result of a linear regression (F_(1,88)_ = 0.0018, p = 0.97) with the shaded area indicating the 95% confidence intervals. Blue points are locus by sample combinations which were not significantly different from the expectations of HWE based on exact tests. Red points are locus by sample combinations which are significantly deviated from the expectations of HWE based on exact tests.

There were deviations from the expectations of HWE in 48% of site by locus comparisons (S1 Fig). These deviations from HWE were attributed to homozygote excess which was observed in 53% of comparisons (S2 Fig). The ubiquity of the homozygote excesses in all sites and in all but one locus does not support the hypothesis of null alleles but rather, indicates potentially high levels of inbreeding [30]. Additionally, we found no relationship between locus specific sample deviations from the expectations of HWE and the percent of individuals that failed to amplify at each locus (F_(1,88)_ = 0.0018, p = 0.97, Fig 2). Additionally, all samples had significant estimates of F_is_ for six or more loci (Table 2). All these results taken together refute the hypothesis of null alleles driving the patterns seen here.

**Table 2:** Inbreeding coefficients for each geographic sample by locus.

	Cope9	Cope12	Cper26	Cper92	Cper99	Cper103	Cper119	Cper163	Cper184	Cper188
TA1	**0.292**	0.090	**0.172**	**0.496**	**0.358**	**0.168**	**0.236**	**0.460**	0.071	**0.169**
TA2	**0.328**	0.004	0.065	**0.539**	**0.444**	**0.343**	**0.432**	**0.657**	-0.037	**0.418**
TA3	**0.219**	**0.444**	0.046	**0.426**	**0.440**	-0.127	**0.252**	**0.606**	-0.119	**0.430**
BBR1	**0.491**	**0.721**	0.098	**0.630**	0.007	0.119	**0.142**	**0.842**	**0.134**	**0.286**
BBR2	**0.330**	**0.670**	0.0091	**0.707**	**0.674**	-0.030	**0.426**	**0.876**	0.010	**0.552**
BBR3	**0.441**	**0.405**	**0.285**	**0.435**	**0.571**	0.064	**0.344**	**0.856**	**0.207**	**0.455**
BC1	**0.338**	**0.229**	-0.218	**0.609**	**0.458**	-0.542	**0.302**	**0.822**	**0.341**	**0.418**
BC2	**0.376**	0.199	**0.210**	**0.395**	0.182	-0.559	**0.232**	**0.590**	0.088	**0.392**
RO1	**0.433**	**0.311**	0.142	0.132	**0.446**	-0.063	**0.148**	**0.694**	**0.100**	-0.066

Bold numbers indicate significant results.

Significant differences in *P*_*D*_ and *F*_*ST*_ were seen in 69% and 97% of the pairwise site comparisons respectively, even between closely spaced sites (ex. TA1 –TA2: ~5 km) (Table 3). Mantel tests for an isolation-by-distance pattern of differentiation were not significant, indicating no correlation between genetic and geographic distance (*P*_*D*_, *r* = 0.22, *P* = 0.19; *F*_*st*_, *r* = 0.31, *P* = 0.14).

**Table 3:** Genetic distance between locations.

	TA1	TA2	TA3	BBR1	BBR2	BBR3	BC1	BC2	R01
TA1	-	**0.316**	0.228	0.228	**0.294**	**0.256**	0.195	0.244	**0.260**
TA2	**0.037**	-	**0.292**	**0.273**	**0.277**	**0.321**	**0.319**	**0.347**	**0.320**
TA3	**0.021**	**0.026**	-	0.225	**0.305**	**0.308**	0.218	0.243	**0.268**
BBR1	**0.016**	**0.020**	**0.019**	-	0.225	0.245	0.247	**0.279**	**0.265**
BBR2	**0.032**	**0.020**	**0.034**	**0.016**	-	**0.256**	**0.320**	**0.333**	**0.327**
BBR3	**0.020**	**0.022**	**0.028**	**0.016**	**0.021**	-	**0.309**	**0.339**	**0.318**
BC1	0.011	**0.031**	**0.016**	**0.021**	**0.042**	**0.030**	-	0.244	**0.279**
BC2	**0.037**	**0.042**	**0.032**	**0.039**	**0.058**	**0.049**	**0.031**	-	**0.282**
R01	**0.028**	**0.026**	**0.025**	**0.020**	**0.037**	**0.025**	**0.027**	**0.038**	-

Pairwise P_D_ (above diagonal) and F_ST_ (below diagonal) with bolded numbers indicating significant values.

**Fig 3:**
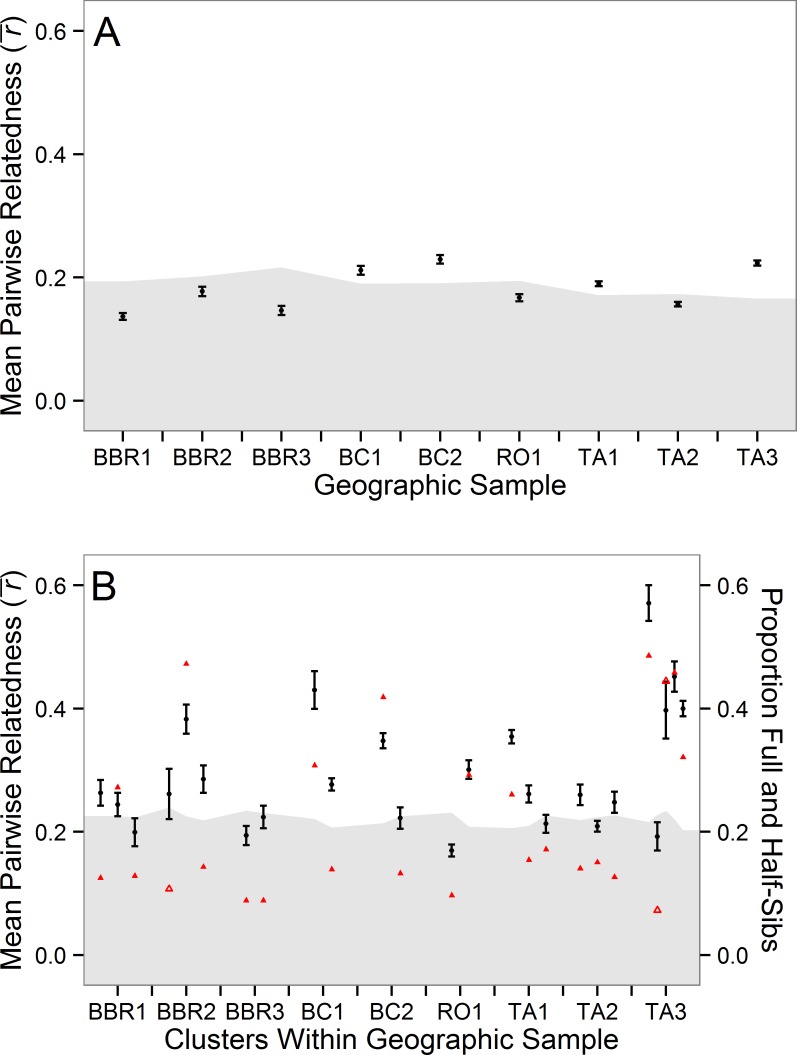
Mean pairwise relatedness ($\bar{r}$) values by geographic sample (A) and cluster (B) with standard errors. The shaded region indicates the area within the 95% confidence intervals calculated using a permutation test with 1,000 iterations. Triangles in 3B indicate proportion of full and half-sibs within the cluster with shaded symbols indicating significantly elevated transitivity when compared to randomly generated networks equivalent to those observed in the clusters.

Within sample mean pairwise relatedness was 34–60% higher than the random expectation in four sites (BC1, BC2, TA1 and TA3; *p* < 0.05, Fig 3A). Within-site clustering analyses found 2 to 5 highly related groups of individuals (8–36 individuals per cluster) within each of the nine geographic samples (Figs 1 and S3). Group relatedness measured within these clusters was found to be 73–314% higher than expected in 68% of all clusters (*p* <0.05, Fig 3B).

The probability of excluding an unrelated individual from a sibship group was 0.998 when all loci are used and 0.927 (95% CI 0.738 to 0.992) when using a random combination of five loci (the minimum number of loci amplified in any individual). The proportion of full- and half-sibs within clusters was calculated to be 22.4±13.8% (S.D.; 3.0±4.4% and 19.3±11.5% respectively) and was significantly greater than clusters of random individuals generated by permutation (*p* <0.05; Table 4; Fig 3B). The cluster networks were found to be composed of a few large groups of siblings and have a mean transitivity of 0.504±0.213 which was significantly greater than graphs of equal order and size generated through the Erdos-Renyi model in all but four clusters (*p* <0.05; Table 4).

**Table 4:** Relatedness and sibship summary of genetic clusters within sample sites.

Site	Cluster	N	$\bar{r}$ ± SE	Possible dyads	Full-Sib dyads	Half-Sib dyads	Sib dyads	Transitivity ± σ
BBR1	G1	17	**0.263 ± 0.021**	136	**3**	**14**	**17**	**0.573 ± 0.481**
BBR1	G2	17	**0.244 ± 0.019**	136	**6**	**31**	**37**	**0.513 ± 0.424**
BBR1	G3	13	0.199 ± 0.023	78	0	**10**	**10**	**0.359 ± 0.461**
BBR2	G1	8	**0.261 ± 0.041**	28	0	**3**	**3**	0.000 ± 0.000
BBR2	G2	14	**0.383 ± 0.024**	91	**11**	**32**	**43**	**0.718 ± 0.395**
BBR2	G3	14	**0.285 ± 0.022**	91	2	**11**	**13**	**0.433 ± 0.421**
BBR3	G1	17	0.194 ± 0.016	136	1	**11**	**12**	**0.431 ± 0.483**
BBR3	G2	17	0.224 ± 0.018	136	0	**12**	**12**	0.206 ± 0.398
BC1	G1	14	**0.430 ± 0.030**	91	**16**	**12**	**28**	**0.750 ± 0.427**
BC1	G2	34	**0.277 ± 0.010**	561	6	**72**	**78**	**0.558 ± 0.368**
BC2	G1	30	**0.348 ± 0.012**	435	**23**	**159**	**182**	**0.770 ± 0.367**
BC2	G2	17	0.222 ± 0.017	136	1	**17**	**18**	**0.329 ± 0.398**
RO1	G1	25	0.169 ± 0.010	300	0	**29**	**29**	**0.461 ± 0.446**
RO1	G2	19	**0.301 ± 0.015**	171	3	**47**	**50**	**0.533 ± 0.438**
TA1	G1	36	**0.354 ± 0.011**	630	9	**155**	**164**	**0.717 ± 0.377**
TA1	G2	23	**0.261 ± 0.014**	253	2	**37**	**39**	**0.607 ± 0.431**
TA1	G3	21	0.213 ± 0.015	210	3	**33**	**36**	**0.354 ± 0.374**
TA2	G1	19	**0.260 ± 0.017**	171	3	**21**	**24**	**0.519 ± 0.487**
TA2	G2	29	0.209 ± 0.009	406	**5**	**56**	**61**	**0.628 ± 0.411**
TA2	G3	23	**0.248 ± 0.017**	253	3	**29**	**32**	**0.424 ± 0.426**
TA3	G1	15	**0.571 ± 0.029**	105	3	**48**	**51**	**0.720 ± 0.416**
TA3	G2	11	0.192 ± 0.023	55	0	**4**	**4**	0.000 ± 0.000
TA3	G3	9	**0.397 ± 0.046**	36	1	**15**	**16**	0.548 ± 0.314
TA3	G4	16	**0.452 ± 0.025**	120	**15**	**40**	**55**	**0.688 ± 0.479**
TA3	G5	34	**0.400 ± 0.012**	561	**11**	**169**	**180**	**0.754 ± 0.307**

Bold values indicate significance based on permutation tests described in methods.

## Discussion

Clusters of highly related individuals were found within geographic samples of *Coryphopterus personatus* (Fig 3B). This explained wide-spread deviations from HWE, genetic differentiation over short distances–as little as 5 km–and the non-equilibrium spatial pattern of genetic differentiation. While we do not have appropriate samples to determine temporal stability of these patterns, all of these results are consistent with CGP. Some geographic samples as a whole showed substantially higher than expected levels of relatedness. However, we found that within each sample there were between 2 and 5 highly related clusters of individuals. These clusters had significantly higher proportions of full- and half-sibs than expected and showed high levels of interconnectedness with a few large groups of related individuals, indicating familial relationships among individuals in these groups. This is unexpected for a species with pelagic larvae where the prevailing paradigm suggests wide dispersal and mixing via physical oceanographic processes and homogenizing the gene pool over large distances.

We propose two mechanisms driving the formation of related groups in this species. First, larvae could remain together in the plankton and settle onto a reef together. There are many potential selective benefits to larvae remaining in a group throughout the pelagic larval phase including predator avoidance [31] and maintenance of position within a food patch [32]. The presence of kin-aggregations may simply reflect the fact that group formation occurs in the egg phase. Consistent with this mechanism, related individuals have been found within a single larval cohort in several other species [7,11]. Given this mechanism, we expect to find individuals of the same cohort within an aggregation to be related to each other, but not related to individuals from other cohorts within the same sample site.

Another mechanism that could explain kin-groups on a coral reef is a lack of larval dispersal. It is possible that larvae do not enter the pelagic environment, remaining on their natal reef. Other marine species that do not have a pelagic larval stage are characterized by low levels of gene flow, frequent population bottlenecks and strong phylogeographic breaks [33]. If *C*. *personatus* has lost their pelagic life stage, we expect a similar genetic pattern. Additionally, we would expect to see highly related individuals across cohorts within a single sample site. Natal recruitment increases the chance of settling in a suitable habitat [34] and, given that *C*. *personatus* needs to feed 2 days post-hatching [35], it is plausible that rather than entering the plankton where food is sparse and patchy [36] larvae could remain on the reef where food is more abundant [37]. However, given the lack of observed isolation by distance between geographic locations this mechanism seems less likely than the possibility of individuals staying together throughout the planktonic phase.

The formation of kin-aggregations is a potentially important evolutionary process due to the likelihood of very little gene flow on extremely small spatial scales and increased rates of inbreeding, potentially leading to greater vulnerability to extirpation [38]. The benefits of kin-aggregations (protection from predation, kin-selection, etc. [39]) may outweigh the costs of inbreeding. The pattern revealed here demonstrates that benthic kin-aggregations are possible in marine species with pelagic larvae and may be more common than previously expected.

## Supporting Information

S1 Fig Deviations from Hardy-Weinberg Equilibrium shown at each geographic sample (rows) for each locus (columns). Significant deviations at a particular sample by locus comparison indicated with a black box.
(TIFF)

S2 Fig Homozygote Excess shown at each geographic sample (rows) for each locus (columns). Significant deviations at a particular sample by locus comparison indicated with a black box.
(TIFF)

S3 Fig Plots showing the number of clusters compared to BIC for each geographic sample. The red points indicate the minimum BIC which was then used as the most likely number of clusters present within the site.
(TIFF)

S1 File Latitude/longitude coordinates for sample locations. (CSV)

S2 File Microsatellite repeat data for individuals with collection location. (CSV)

S1 Table Table of proportion of individuals which failed to amplify at each site/locus combination. (XLSX)
