# Supplementary figures and images for: Kin-Aggregations Explain Chaotic Genetic Patchiness, a Commonly Observed Genetic Pattern, in a Marine Fish
Source: PLoS One. 2016 Apr 27;11(4):e0153381. doi: 10.1371/journal.pone.0153381 (PMC4847911; doi:10.1371/journal.pone.0153381)

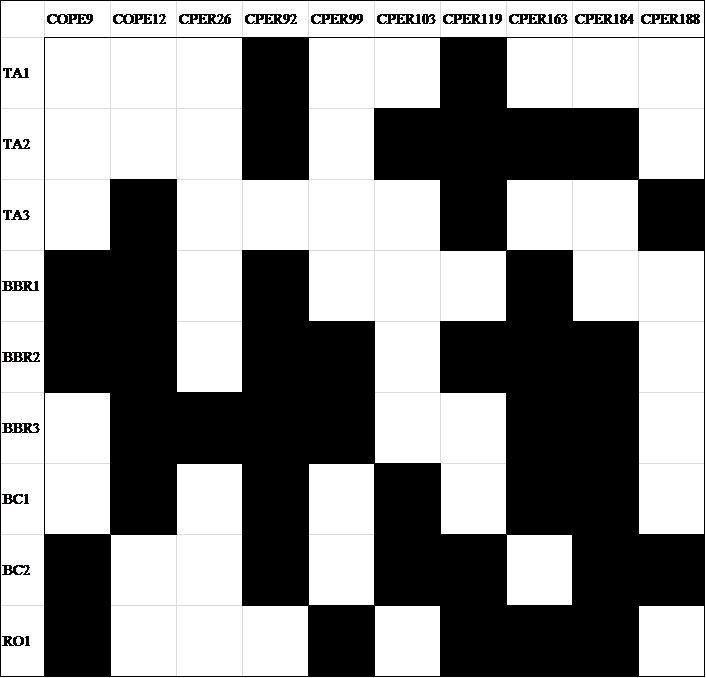

Supplement: S1 Fig — Significant deviations at a particular sample by locus comparison indicated with a black box. (TIFF) [file pone.0153381.s001.tiff]

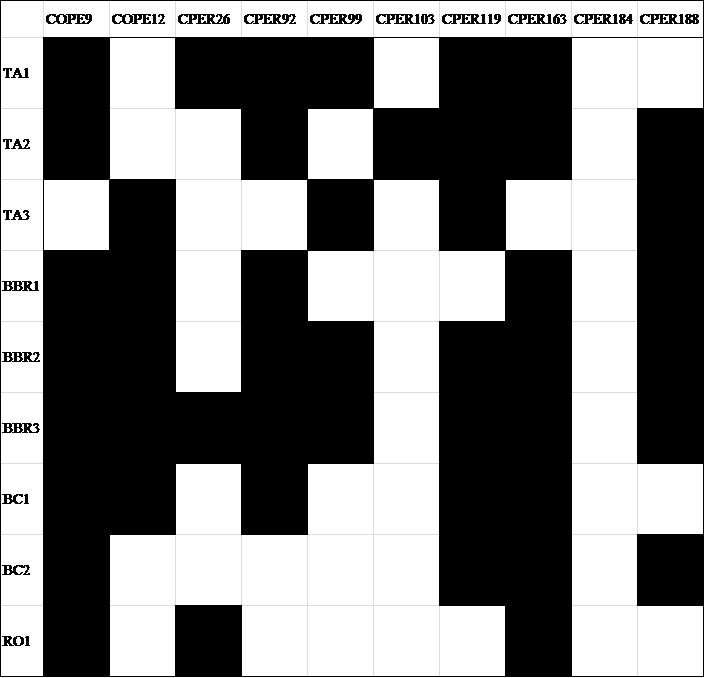

Supplement: S2 Fig — Significant deviations at a particular sample by locus comparison indicated with a black box. (TIFF) [file pone.0153381.s002.tiff]

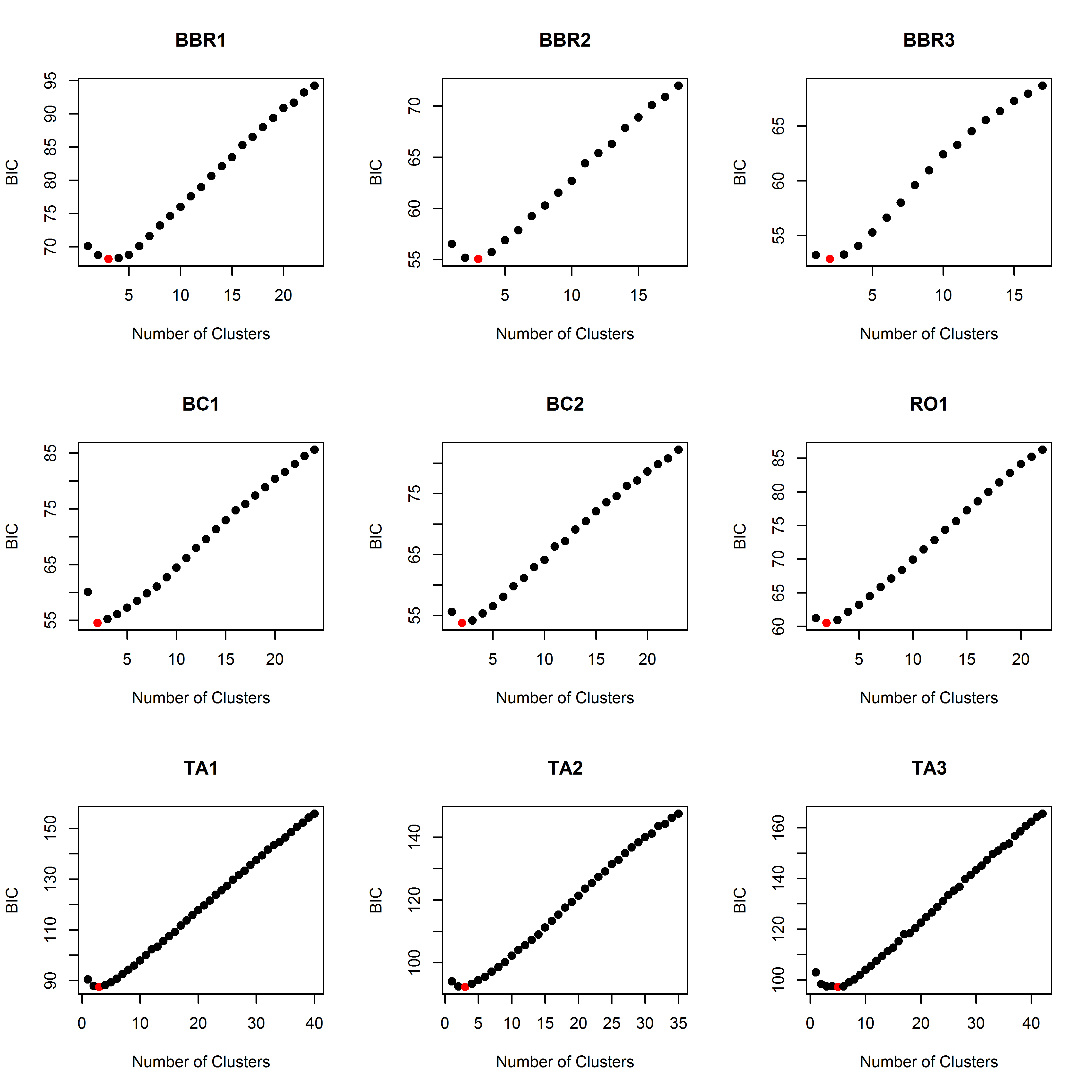

Supplement: S3 Fig — The red points indicate the minimum BIC which was then used as the most likely number of clusters present within the site. (TIFF) [file pone.0153381.s003.tiff]
